# Mini-donor nephrectomy: A viable and effective alternative

**DOI:** 10.4103/0970-1591.60464

**Published:** 2010

**Authors:** Sandeep Guleria

**Affiliations:** Department of Surgery, All India Institute of Medical Sciences, Ansari Nagar, New Delhi-110 029, India

**Keywords:** Laparoscopic, mini-donor, nephrectomy

## Abstract

Live kidney donation is an excellent way of increasing the donor pool. The introduction of the laparoscopic donor nephrectomy has resulted in an increase in live organ donation in the western hemisphere. There is no data on its impact on organ donation in India. However attractive as it may seem, the procedure is associated with a definite learning curve and does compromise donor safety. The procedure is also expensive in terms of the equipment required. The mini-donor nephrectomy is an excellent alternative, has no learning curve and is ideally suited for donors in India who have a low BMI. The procedure is also relatively inexpensive. We are in need of a donor registry rather than reports from single institutions to fully evaluate the risks and benefits of both procedures.

## INTRODUCTION

Live donor transplantation provides a significant advantage when compared with cadaveric renal transplantation in terms of improved patient and graft survival. With excellent one and five-year graft survivals, the disparity between demand and supply has grown. Most transplant units have looked at their live donors to increase the supply of organs. The driving force for live related renal donation has been an excellent track record of donor safety and the optimal quality of the organ that has been procured.

The major disincentive to live renal donation organ is the length of hospital stay, postoperative pain and the time taken to get back to full activity.[1] Laparoscopic donor nephrectomy has had a considerable impact on renal donation in the western hemisphere. Live donors now account for more than 50% of the kidneys transplanted in the western hemisphere.

## OPEN DONOR NEPHRECTOMY AND LAPAROSCOPIC DONOR NEPHRECTOMY

There is little doubt that there is significant scope for improvement with the conventional muscle cutting rib resecting incision, which leads to considerable pain and muscle weakness resulting in a bulge in the postoperative period. Both laparoscopic and the mini-donor nephrectomy are effective alternatives. Laparoscopic donor nephrectomy represents a significant surgical advance for the live renal donor. It is associated with a smaller scar-less postoperative pain and quicker recovery. Most donors are able to get back to their original jobs within two weeks.[1–3] The surgery is also associated with less pain and more appealing to the lay public.

A review of the existing literature reveals that the subject of live donor nephrectomy is a seat of under-reporting and under estimation of complication. The advent of the laparoscopic donor nephrectomy has resulted in most centers in India critically evaluating the service that they offer their living donors. It is important to remember that the donor nephrectomy has two vital components the safety of the donor and the optimal condition of the organ being harvested. The flank approach to donor nephrectomy has stood the test of time with regards to both .The operation is safe and provides a good quality organ. However, it is associated with significant morbidity in terms of loin pain, structural disability and cosmesis, which may occur in 7 to 9% of patients.

In a study by Shaffer *et al*.[4] of 201consecutive donors, bleeding was encountered in one patient, pneumo-thorax in two, wound infection in two and pneumonia in two. Only one patient developed an incisional hernia. Complications in laparoscopic donor nephrectomy are serious. Retroperitoneal hematoma was encountered in four patients, splenic injury in two patients, bowel injury in three patients and renal vein tear in one patient. Six patients required a blood transfusion.^[56]^

There is little or no data on its impact in transplantation in India. Laparoscopic donor nephrectomy is associated with a learning curve and is associated initially with significant morbidity. In terms of donor safety in laparoscopic donor nephrectomy at least nine peri-operative deaths have been reported with and at least 15 graft losses directly related to the surgical technique of laparoscopic donor nephrectomy were found.^[64]^

In India, cadaver organ donation accounts for an insignificant number of renal donations. Almost all donations are from live donors. These are either related in the vast majority of cases or authorized as per the Human Organ Transplant Act. The commonest donor is usually the female sex and is usually the mother, wife or sister. Most of these donations are to bread winners of the family to whom the entire economic survival of the family is linked to. Also, vast majority of these donors are unemployed. For them, the most important factor is the quality of organ harvested and the quality of life that recipient enjoys to make him the bread winner again.

### Pneumo-peritoneum in laparoscopic donor nephrectomy

The pneumo-peritoneum that is created during laparoscopic donor nephrectomy may be detrimental on both pulmonary function and vascular perfusion.[7] The increased intra-abdominal pressure may result in decreased renal vein blood flow with reduced cortical and medullary perfusion which may result in delayed function. Rigorous over-hydration may correct this. In India, where most of the living donors are old, this over hydration may have a detrimental effect on donor cardiac function. Indeed there have been reports in literature of donors getting pulmonary edema.

### Side selection issues in laparoscopic donor nephrectomy

The live donor is sacred to the renal transplant program. Most open donor programs leave the better functioning kidney behind. This has resulted in the right kidney being harvested in approximately 22% to 35% of patients. The right kidney is more difficult to harvest laparoscopically and this has resulted in the right kidney being harvested in only 2% to 5% of donors.[8] Laparoscopic donor nephrectomy thus compromises a fundamental principle of live organ donation. The easier side organ to retrieve laparoscopically takes priority over a fundamental principle of organ donation.

### Urological complications in laparoscopic donor nephrectomy

Most centers that embarked on a laparoscopic donor nephrectomy program reported on an initial incidence of urological complications to be as high as 10.5%. In open donor nephrectomy, the complications are less than 2%. In laparoscopy, this incidence has declined with increasing experience and appreciation of the fact that the gonadal vein should be harvested with the ureter.[8]

## COST AND LAPAROSCOPIC DONOR NEPHRECTOMY

The economic considerations in laparoscopic donor nephrectomy can be considerable. In the western hemisphere the cost of disposables required for laparoscopic surgery is $2251 as compared to $812 for an open donor nephrectomy.[9] The big advantage in the western hemisphere is that the laparoscopic donor goes home earlier resulting in saving in terms of bed occupancy. In India where hospital stay is fairly cheap and where the family has sold every piece of property for recurring costs of immunosuppressants, the saving in terms of disposables can be considerable.

The issue of securing the pedicle in a laparoscopic donor nephrectomy has aroused considerable controversy. There are at least two reports in literature of hem-o-lock clips slipping in the donor leading to exsanguinating hemorrhage. [14] It is now recommended that the some form of tissue fixation using a vascular stapler should be used. This can lead to considerable cost escalation as donor safety should be sacrosanct.

## MINI-DONOR NEPHRECTOMY VS. LAPAROSCOPIC DONOR NEPHRECTOMY

At our center, we have explored mini-donor nephrectomy and laparoscopic donor nephrectomy as an option for our live related renal donors. The mini donor nephrectomy procedure consists of a non rib resecting flank incision above the eleventh rib which is extended depending upon the position of the kidney. The ureter is mobilized with the gonadal vein and the artery and nein are dissected. Two narrow based retractors are used and the artery and vein are ligated. The incision is closed inlayers with 4-0 monocryl applied to the skin.

Our initial results on 78 donors with a mean age of 40.98 years and a mean weight of 63.66 Kg revealed an incision length of 9.2 cm and a time to harvest of 45.15 minutes. The mean post-operative stay was 2.26 days and no kidney was lost nor any major complication encountered in the donor. [10] We commenced a laparoscopic donor nephrectomy program in 2007 and have currently done more than one hundred and twenty six such procedures. We recently conducted a randomized trial to compare the laparoscopic donor nephrectomy with the mini donor nephrectomy in our settings. [11] We are a State-funded apex teaching institution of the government and all cost are borne by the state except the recurring cost of immunosuppression which the patient has to buy.

Sixty consecutive donors were randomized into two groups. Group 1 underwent a laparoscopic donor nephrectomy while group II underwent an open donor nephrectomy. Laparoscopic donor nephrectomy was carried out using a standard four port approach and the kidney was removed manually. The technique of the mini-donor nephrectomy has already been described.

Both groups were evaluated with regard to operative time, postoperative stay and the visual analogue pain score at the time of discharge. The graft function was also evaluated at one month. Both groups were also evaluated to the resumption of normal diet and the BMI also noted [Table 1].

**Table 1:** Comparison of laparoscopic and open donor nephrectomy

	Mini-donor (n=30)	Laparoscopic donor (n=30)
Age (Mean Years)	47.3	40.6
Sex distribution (M: F)	6:24	4:26
BMI	19.9	21.9
Operating time (minutes)	55.23	132.34
Resumption of normal feeding	1	4
Post operative stay (days)	3	4
Visual Pain Score on day 7	4	6

No graft or donor morbidity occurred in either group. Ileus was a significant problem in the laparoscopic group and this lead to a significant number of donors complaining of discomfort and pain in the post –operative period. It was primarily because of this discomfort and ileus that the laparoscopic group had a longer postoperative stay then the mini-donor group. We thus concluded that in our setting where most of the donors have a low BMI the mini-donor nephrectomy may be a more viable option in terms of donor safety, economics and recovery.

Nanidis[12]*et al*. in a recent meta analysis comparing laparoscopic versus open donor nephrectomy evaluated seventy three studies and concluded that though open donor nephrectomy was associated with a shorter post-operative and warm ischemia times patients undergoing laparoscopic donor nephrectomy may benefit from a shorter hospital stay and faster return to work without compromising graft function.

In another recent article comparing the mini-incision muscle splitting (MIDN) open approach to the laparoscopic donor nephrectomy (LDN) Kok *et al*. [13] found that MIDN resulted in shorter warm ischemia time and operation time. More over MIDN was cheaper (328 versus 1784 Euros) and had lesser complications both in terms of major post operative and major intra operative as compared to the laparoscopic approach. However, MIDN donors stayed a day more than laparoscopic donors. One-year graft survival was 100% following MIDN and 86% in the LDN group. They concluded that both approaches can be used to expand living donor programs.

## CONCLUSION

Laparoscopic donor nephrectomy remains a procedure in evolution. The high costs and the training required have been major deterrents to its widespread use in India. We are in need of a live organ donor registry to determine the combined experience of complications and long term outcomes, rather than short term reports from single institutions. However, with requisite training it remains an attractive alternative for renal donation.

## References

[1] Ratner LE, Kavoussi LR, Sroka M, Hiller J, Weber R, Schulam PG (1997). Laparoscopic assisted live donor nephrectomy--a comparison with the open approach. Transplantation.

[2] Kuo PC, Johnson LB (2000). Laparoscopic donor nephrectomy increases the supply of living donor kidneys: a center-specific microeconomic analysis. Transplantation.

[3] Ratner LE, Buell JF, Kuo PC (2000). Laparoscopic donor nephrectomy: pro. Transplantation.

[4] Shaffer D, Sayhoun AI, Madras P, Monaco AP (1998). Two hundred one consecutive living-donor nephrectomies. Arch Surg.

[5] Montgomery RA, Kavoussi LR, Su L, Sinkov V, Cohen C, Maley WR (2001). Improved recipient results after 5 years of performing laparoscopic donor nephrectomy. Transplant Proc.

[6] Shokeir AA (2007). Open versus laparoscopic live donor nephrectomy: a focus on the safety of donors and the need for a donor registry. J Urol.

[7] Nogueira JM, Cangro CB, Fink JC, Schweitzer E, Wiland A, Klassen DK (1999). A comparison of recipient renal outcomes with laparoscopic versus open live donor nephrectomy. Transplantation.

[8] Barry JM (2000). Laparoscopic donor nephrectomy: con. Transplantation.

[9] Guleria S (2003). Is minimally invasive donor nephrectomy the future?. Transplant Proc.

[10] Guleria S, Aggarwal S, Mandal S, Singh P, Mehta SN, Aggarwal SK (2003). The mini-donor nephrectomy: a viable option. Transplant Proc.

[11] Guleria S, Kumar R, Reddy VS, Jain S, Aggarwall S, Mahajan S (2008). Is Laparoscopic donor nephrectomy really the way ahead?. Transplantation ;:2S.

[12] Nanidis TG, Antcliffe D, Kokkinos C, Borysiewicz CA, Darzi AW, Tekkis PP (2008). Laparoscopic versus open live donor nephrectomy in renal transplantation a meta analysis. Ann Surg.

[13] Kok NF, Lind MY, Hansson BM, Pilzecker D, Mertens zur Borg IR, Knipscheer BC (2006). Comparison of laparoscopic and mini incision open donor nephrectomy: single blind, randomised controlled clinical trial. BMJ.

[14] Dekel Y, Mor E (2008). Hem-o-lok clip dislodgment causing death of the donor after laparoscopic living donor nephrectomy. Transplantation.

